# Recurrent and De Novo Toxoplasmosis Retinochoroiditis following Coronavirus Disease 2019 Infection or Vaccination

**DOI:** 10.3390/vaccines10101692

**Published:** 2022-10-10

**Authors:** Mélanie Hébert, Soumaya Bouhout, Julie Vadboncoeur, Marie-Josée Aubin

**Affiliations:** 1 Department of Ophthalmology, Université Laval, Quebec, QC G1V 0A6, Canada; 2 Department of Ophthalmology, Université de Montréal, Montreal, QC H3T 1J4, Canada; 3 Ophthalmology Department, Centre Universitaire d’Ophtalmologie (CUO), Centre Intégré Universitaire de Santé et de Services Sociaux de l’Est-de-l’Île-de-Montréal—Hôpital Maisonneuve-Rosemont, Montreal, QC H1T 2M4, Canada; 4 Department of Social and Preventive Medicine, School of Public Health, Université de Montréal, Montreal, QC H3T 1J4, Canada

**Keywords:** coronavirus disease 2019, vaccination, mRNA vaccine, SARS-CoV-2, toxoplasmosis retinochoroiditis, inflammation, antibiotic prophylaxis, ophthalmic adverse events, COVID-19 vaccination

## Abstract

This study reports three cases of toxoplasmosis retinochoroiditis following coronavirus disease 2019 (COVID-19) infection or vaccination from the national Canadian COVID-19 Eye Registry between December 2020 and September 2021. A 56-year-old male presented 15 days after a positive COVID-19 test with toxoplasmosis retinochoroiditis. He later relapsed 8 days following a first Pfizer-BioNTech vaccine dose. Two patients presented with toxoplasmosis retinochoroiditis following COVID-19 vaccination: A 58-year-old female presenting 4 days following a first Pfizer-BioNTech vaccine dose with anterior uveitis and a posterior pole lesion discovered 3 months later and a 39-year-old female presenting 17 days after a first Moderna vaccine dose. Resolution was achieved with oral clindamycin, oral trimethoprim/sulfamethoxazole, and topical prednisolone acetate 1%. Patients were offered prophylactic trimethoprim/sulfamethoxazole for subsequent doses without relapse. Following COVID-19 infection or vaccination, patients may be at risk for toxoplasmosis retinochoroiditis. Prophylactic antibiotics for future doses may be offered to patients with known ocular toxoplasmosis to prevent recurrence.

## 1. Introduction

Toxoplasmosis, caused by the protozoan parasite *Toxoplasma gondii*, is an opportunistic infection which can affect healthy adults but targets immunosuppressed people, causing more severe disease [1]. When this affects the eye, ocular toxoplasmosis can cause severe vision loss especially with macular involvement [1,2]. Many causes of relative immune suppression have reportedly caused reactivations of ocular toxoplasmosis, such as dexamethasone intravitreal implants [3] and azathioprine treatment for inflammatory bowel disease [4].

Another emerging cause of relative immunosuppression is the coronavirus disease 2019 (COVID-19). It triggers dysfunction and reduced numbers of T cells, natural killer cells, monocytes, and dendritic cells [5]. This may explain reports of de novo infections or reactivations following COVID-19 disease, including herpes simplex keratitis [6] and herpes zoster ophthalmicus even in children [7].

Another possible immune modulator is COVID-19 vaccination. These were shown to be safe and effective [8,9,10], allowing for the reduction in virus transmission [11,12]. However, these have also been associated with autoimmune and infectious diseases, such as graft rejections [13] and herpes zoster ophthalmicus [14].

Changes in immunity related to both COVID-19 infection and vaccination may increase the risk of ocular toxoplasmosis relapse. Three cases following COVID-19 vaccination have recently been reported [15]. This case series aims to report three cases of new or recurrent toxoplasmosis retinochoroiditis following COVID-19 infection or vaccination.

## 2. Methods

This study reports results from the COVID-19 Eye Registry (COVER). COVER is a national Canadian registry recording ocular manifestations following COVID-19 infection or vaccination [16]. Patients who were reported between December 2020 and September 2021 with new or recurrent toxoplasmosis retinochoroiditis were presented. Basic demographic data, type of vaccine (i.e., Moderna Spikevax^®^ (ModernaTX, Inc., Cambridge, MA, USA), Pfizer-BioNTech Comirnaty^®^ (BioNTech Manufacturing GmbH, Mainz, Germany), or AstraZeneca Vaxzevria^®^ (AstraZeneca BioPharmaceuticals, Cambridge, England)), timing of presentation relative to COVID-19 infection or vaccination, clinical presentation, management, and final visual acuity (VA) are reported.

## 3. Results

Three eyes of three patients were reported. Of these, a patient presented with de novo toxoplasmosis retinochoroiditis following COVID-19 infection followed by reactivation after a first dose of COVID-19 vaccine. Two other patients presented with unilateral panuveitis and active chorioretinal lesions following COVID-19 vaccination.

### 3.1. Case 1

**Figure 1:**
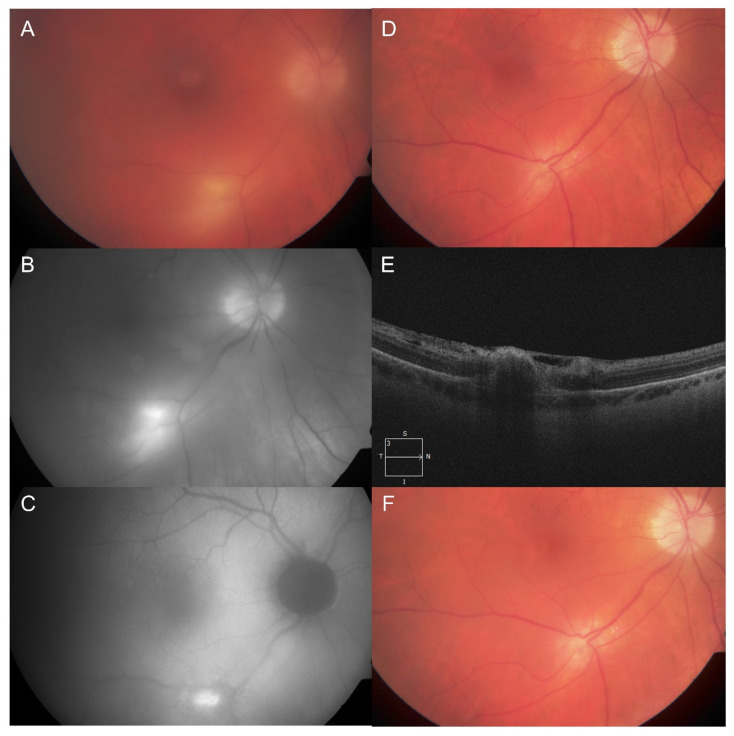
Case 1 with active toxoplasmosis retinochoroiditis following a diagnosis of coronavirus disease 2019 (COVID-19) and recurrence after COVID-19 vaccination. (**A**) Color fundus photography showing a single, white, deep chorioretinal lesion along the inferior arcade with vitritis at initial presentation. (**B**) Initial red-free monochromatic fundus photograph showing the same lesion which becomes more apparent. (**C**) Initial fundus autofluorescence showing a hyperautofluorescent lesion with distinct borders corresponding to the retinochoroiditis. (**D**) Four months after presentation, the lesion is now cicatricial without vitritis with (**E**) a horizontal optical coherence tomography scan through the lesion. (**F**) The patient had a recurrence 1 month later, 8 days after his first COVID-19 vaccine dose.

A 56-year-old male known for high blood pressure and oral herpes simplex virus, with no history of toxoplasmosis, presented with hypertensive anterior uveitis in his right eye (OD). Initial VA was 20/40-2 and dilated fundus examination (DFE) at the time did not show any chorioretinal lesion. He was found to be COVID-19 positive 10 days later. At follow-up 15 days after his positive COVID-19 test, his VA had decreased to 20/70 and repeat DFE showed a unilateral white chorioretinal lesion in the posterior pole without pigmentary or atrophic scarring (Figure 1) associated with vitritis, consistent with ocular toxoplasmosis. Anti-toxoplasmosis immunoglobulin (Ig) G was positive (97.80 g/L), while anti-toxoplasmosis IgM was negative when requested at the follow-up visit. Moreover, the patient tested negative for syphilis but positive for HLA-B27. Resolution was achieved with a course of topical prednisolone acetate 1% with oral clindamycin 450 mg three times daily and trimethoprim/sulfamethoxazole 800/160 mg twice daily over 2 months. One month following the end of treatment, VA was 20/20, no inflammation was observed, and the chorioretinal lesions were cicatricial and inactive. He later presented a retinochoroiditis relapse 8 days following a first dose of Pfizer-BioNTech vaccine, which resolved with the same therapeutic regimen.

A prophylactic course of trimethoprim/sulfamethoxazole 800/160 mg three times per week was started 2 days before his second dose and continued over 2 weeks. No recurrence was observed. He was not seen in clinic prior to his third booster dose to receive a prophylactic prescription. Nevertheless, no recurrence was detected when assessed 1 week later. His final VA was 20/25-1.

### 3.2. Case 2

**Figure 2:**
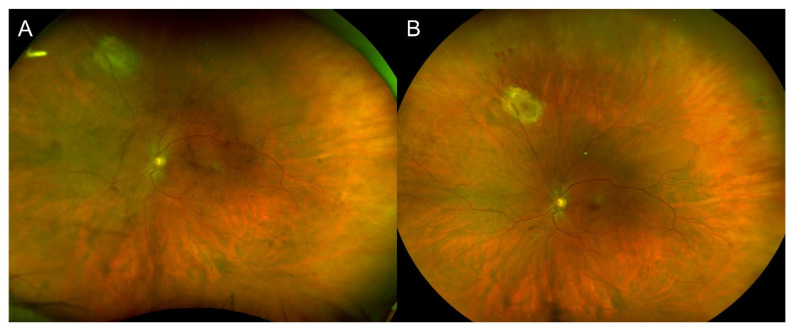
Case 2 with active toxoplasmosis retinochoroiditis 3 months after a first dose of COVID-19 vaccination. (**A**) Fundus photography showing the initial elevated, yellow whitish lesion with surrounding pigmentary changes. (**B**) Six weeks later, the main lesion became cicatricial.

A 58-year-old female known for high blood pressure, dyslipidemia, type II diabetes mellitus, and fibromyalgia was previously treated for anterior uveitis in her left eye (OS) by an optometrist 4 days following her first dose of Pfizer-BioNTech vaccine with topical prednisolone acetate 1% over 1 month. It is unknown whether DFE was performed initially and whether signs of vitritis or chorioretinal lesions were already present. Three months post-vaccination, she was referred to our service for increasing floaters, discomfort, and VA of 20/50. Examination revealed panuveitis with posterior hyaloid precipitates and a slightly elevated, yellow whitish lesion with surrounding pigmentary changes (Figure 2A). Serological testing revealed positive anti-toxoplasmosis IgG (18.80 g/L) and negative anti-toxoplasmosis IgM. Other uveitis testing was negative, including HLA-B27, antinuclear antibodies, anti-double stranded DNA, extractable nuclear antigen antibodies, antineutrophil cytoplasmic antibodies, Lyme, syphilis, and QuantiFERON-TB Gold. She was treated with topical prednisolone acetate 1% four times daily tapered over a month, 6 weeks of trimethoprim/sulfamethoxazole 800/160 mg twice daily, and a short course of oral prednisone starting at 40 mg daily tapered over 5 weeks. Six weeks after the treatment started, there was no intraocular inflammation, the chorioretinal lesion was cicatricial (Figure 2B), and final VA was 20/20. The patient received the same prophylactic trimethoprim/sulfamethoxazole regimen as Case 1 for her second vaccine doses, but not for her third dose, and she did not relapse in both instances.

The patient was not known to have had a previous COVID-19 infection prior to their vaccination or during follow-up. At follow-up, she later received a dose of pneumococcal polysaccharide vaccine (Pneumovax^®^ 23, Merck & Co., Inc., Kenilworth, NJ, USA) and had a recurrence of retinochoroiditis that responded to the same treatment of prednisolone.

### 3.3. Case 3

**Figure 3:**
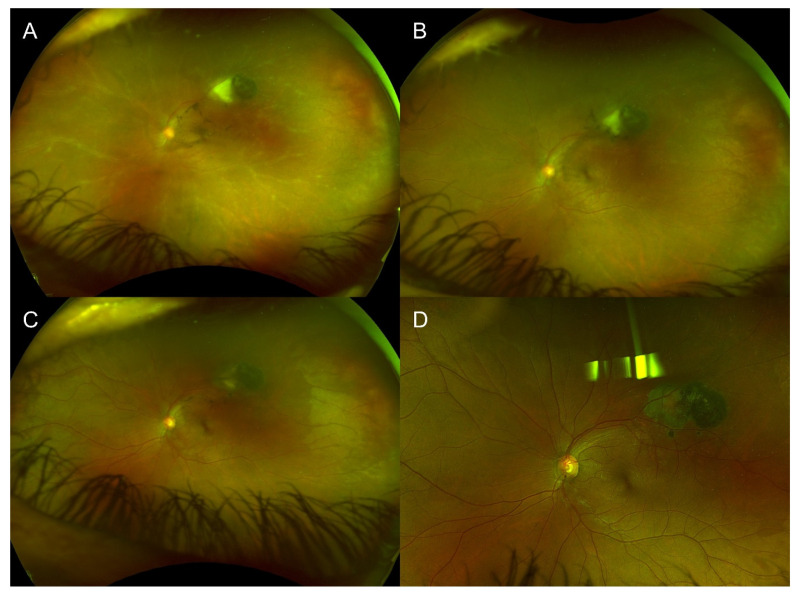
Case 3 with a previous, cicatricial toxoplasmosis scar with recurrence of active toxoplasmosis retinochoroiditis 17 days after COVID-19 vaccination. (**A**) Fundus photography showcasing the old toxoplasmosis scar with an area of active retinochoroiditis adjacent to it. There is diffuse sheathing consistent with vasculitis. (**B**) Three weeks later, the new area of retinochoroiditis has decreased in size and activity as did the vasculitis. (**C**) At seven weeks, there was still an area of activity within the chorioretinal scar, thus the treatment was further extended. (**D**) At final follow-up 4 months later, the area had scarred, and no inflammation was observed.

A 39-year-old female presented 17 days after a first dose of Moderna vaccine with floaters and VA of 20/100. This patient was known to the center’s uveitis service for toxoplasmosis retinochoroiditis since 2011 with no relapse since 2016 without prophylaxis and was otherwise healthy. Her initial exam showed panuveitis with an active fundus lesion around the known chorioretinal scar (Figure 3). She was treated with topical prednisolone acetate 1%, oral clindamycin 600 mg three times daily and trimethoprim/sulfamethoxazole 800/160 mg twice daily over 6 weeks, and a short course of oral prednisone starting at 10 mg with a taper over 4 weeks. She received her second dose of Moderna 12 weeks after presentation while still under trimethoprim/sulfamethoxazole 800/160 mg twice daily without recurrence. At her last follow-up 2 months later, the final VA was 20/20 and the lesion was cicatricial 4 months after the initial presentation. She was offered the same prophylactic trimethoprim/sulfamethoxazole regimen as Case 1 in the event of a third booster dose. The patient was not known to have had a previous COVID-19 infection prior to their vaccination or during follow-up.

## 4. Discussion

We report one case of toxoplasmosis retinochoroiditis following COVID-19 infection with recurrence after COVID-19 vaccination, as well as two cases of toxoplasmosis retinochoroiditis following COVID-19 vaccination. Both Cases 1 and 3 had recurrences of ocular toxoplasmosis within 2–3 weeks following COVID-19 vaccination. Both had previous episodes of toxoplasmosis retinochoroiditis (after COVID-19 infection in Case 1), which may explain the rapid reactivation following vaccination. In Case 2, anterior uveitis was initially diagnosed 4 days after vaccination. As an outside optometrist assessed her, it is unknown whether DFE may have revealed vitritis and retinochoroidal involvement. This patient also recurred following pneumococcal polysaccharide vaccine.

COVID-19 infection disrupts the function and number of T cells, natural killer cells, monocytes, and dendritic cells [5]. This may increase ocular toxoplasmosis reactivation risk as the principal cytokine responsible for the response against *T. gondii* is interferon (IFN)-γ. It is produced by CD4 and CD8 positive T cells, natural killer cells, and neutrophils through a trigger by interleukin 12, produced by dendritic cells [1]. Production of IFN-γ is reduced in active COVID-19 infection [5]. In IFN-γ deficient mice, the extent of toxoplasmosis is more severe with cerebral dissemination and occasionally more severe ocular toxoplasmosis [17]. In humans, serum IFN-γ levels are lower in patients with ocular toxoplasmosis reactivations and old toxoplasmosis scars [18]. Decreased aqueous humor IFN-γ is also associated with increased risk of severe ocular complications and longer time to recovery from reactivations [18]. This is consistent with the fact that various polymorphisms of the *IFNG* gene modify chorioretinal scar severity [1].

The pathophysiology for ocular toxoplasmosis reactivation following COVID-19 vaccination is less clear. The Spike protein targeted by all currently available vaccines can produce robust CD4 and CD8 positive T cell responses [19]. This in turn should produce IFN-γ which should also be efficient against *T. gondii* as part of the innate and early adaptive immune response. This production of IFN-γ is important in protecting against COVID-19, since patients with deficient IFN-γ do not mount an appropriate defense against COVID-19 in the early phases of the disease, leading to more severe illness [19]. However, whether there may be a phenomenon of immune modulation responsible for decreased protection against toxoplasmosis in the immediate post-vaccination period remains to be studied. Moreover, this holds true for other vaccines, such as in Case 2 where the patient experienced a second reactivation after pneumococcal polysaccharide vaccination, which may suggest an underlying immune reaction to vaccinations in general, and not specifically to COVID-19, in susceptible patients. Additionally, with the breadth of the vaccination campaigns around the world, it is also possible that ocular findings after vaccination may occur coincidentally without a true association.

Whether patients known for ocular toxoplasmosis might require prophylactic antibiotics for COVID-19 vaccination is debatable. Antibiotic treatments for toxoplasmosis include combinations of pyrimethamine, sulfadiazine, and leucovorin or trimethoprim-sulfamethoxazole [20,21]. For prophylaxis, trimethoprim-sulfamethoxazole is the preferred option [20]. There is a lack of studies that provide level I evidence to support the antibiotics treatment of toxoplasmosis retinochoroiditis, as it does not seem to reduce lesion size or improve visual outcomes [21,22]. However, there does seem to be a reduced risk of reactivation with prophylactic treatment [21,22], which could be of particular interest in the setting of COVID-19 vaccination in patients with known toxoplasmosis retinochoroiditis, especially in those with posterior pole lesions. This remains to be studied but could be discussed with patients as possible management options in the absence of more evidence.

### Limitations

There is no definitive causal relationship between COVID-19 infections or vaccines and toxoplasmosis retinochoroiditis. The temporal relationship makes this plausible although it could be a coincidence given the billions of vaccine doses administered worldwide. Since recurrences of ocular toxoplasmosis are treatable and preventable, it should not deter vaccination efforts. Similarly, we could not be certain of the temporality of Case 2 wherein a reliable DFE was not performed despite increasing floaters and discomfort between 4 days and 3 months post-vaccination.

## 5. Conclusions

In conclusion, following COVID-19 infection or vaccination, patients may be at risk for new or relapsing toxoplasmosis retinochoroiditis, possibly due to changes in immune modulation. Physicians should be aware of this and may propose prophylactic antibiotics for future vaccine doses in patients known for ocular toxoplasmosis, especially those with vision-threatening posterior pole lesions.

## Data Availability

The data are available upon reasonable request made to the data access committee of the COVID-19 Eye Registry.

## References

[1] Smith J.R., Ashander L.M., Arruda S.L., Cordeiro C.A., Lie S., Rochet E., Belfort R., Furtado J.M. (2021). Pathogenesis of ocular toxoplasmosis. Prog. Retin. Eye Res..

[2] Vishnevskia-Dai V., Achiron A., Buhbut O., Berar O.V., Musika A.A., Elyashiv S.M., Hecht I. (2020). Chorio-retinal toxoplasmosis: Treatment outcomes, lesion evolution and long-term follow-up in a single tertiary center. Int. Ophthalmol..

[3] Olson D.J., Parhiz A.T., Wirthlin R.S. (2016). Reactivation of Latent Toxoplasmosis Following Dexamethasone Implant Injection. Ophthalmic Surg. Lasers Imaging Retin..

[4] Puga M., Carpio D., Sampil M., Zamora M.J., Fernandez-Salgado E. (2016). Ocular Toxoplasmosis Reactivation in a Patient With Inflammatory Bowel Disease Under Treatment With Azathioprine. J. Clin. Gastroenterol..

[5] Zhou R., To K.K., Wong Y.C., Liu L., Zhou B., Li X., Huang H., Mo Y., Luk T.Y., Lau T.T. (2020). Acute SARS-CoV-2 Infection Impairs Dendritic Cell and T Cell Responses. Immunity.

[6] Majtanova N., Kriskova P., Keri P., Fellner Z., Majtan J., Kolar P. (2021). Herpes Simplex Keratitis in Patients with SARS-CoV-2 Infection: A Series of Five Cases. Medicina.

[7] Nofal A., Fawzy M.M., Sharaf ELDeen S.M., El-Hawary E.E. (2020). Herpes zoster ophthalmicus in COVID-19 patients. Int. J. Dermatol..

[8] Polack F.P., Thomas S.J., Kitchin N., Absalon J., Gurtman A., Lockhart S., Perez J.L., Marc G.P., Pérez G., Moreira E.D. (2020). Safety and Efficacy of the BNT162b2 mRNA Covid-19 Vaccine. N. Engl. J. Med..

[9] Baden L.R., El Sahly H.M., Essink B., Kotloff K., Frey S., Novak R., Diemert D., Spector S.A., Rouphael N., Creech C.B. (2021). Efficacy and Safety of the mRNA-1273 SARS-CoV-2 Vaccine. N. Engl. J. Med..

[10] Voysey M., Clemens S.A., Madhi S.A., Weckx L.Y., Folegatti P.M., Aley P.K., Angus B., Baillie V.L., Barnabas S.L., Bhorat Q.E. (2021). Safety and efficacy of the ChAdOx1 nCoV-19 vaccine (AZD1222) against SARS-CoV-2: An interim analysis of four randomised controlled trials in Brazil, South Africa, and the UK. Lancet.

[11] Amit S., Regev-Yochay G., Afek A., Kreiss Y., Leshem E. (2021). Early rate reductions of SARS-CoV-2 infection and COVID-19 in BNT162b2 vaccine recipients. Lancet.

[12] Nordström P., Ballin M., Nordström A. (2021). Association Between Risk of COVID-19 Infection in Nonimmune Individuals and COVID-19 Immunity in Their Family Members. JAMA Intern. Med..

[13] Abousy M., Bohm K., Prescott C., Bonsack J.M., Rowhani-Farid A., Eghrari A.O. (2021). Bilateral EK Rejection After COVID-19 Vaccine. Eye Contact Lens..

[14] Papasavvas I., de Courten C., Herbort C.P. (2021). Varicella-zoster virus reactivation causing herpes zoster ophthalmicus (HZO) after SARS-CoV-2 vaccination–report of three cases. J. Ophthalmic Inflamm. Infect..

[15] Bolletta E., Iannetta D., Mastrofilippo V., De Simone L., Gozzi F., Croci S., Bonacini M., Belloni L., Zerbini A., Adani C. (2021). Uveitis and Other Ocular Complications Following COVID-19 Vaccination. J. Clin. Med..

[16] Hébert M., Buys Y.M., Damji K.F., Yin V.T., Aubin M.J. (2021). Data reporting in ophthalmology during COVID-19 pandemic: Need for a Canadian registry. Can. J. Ophthalmol..

[17] Jones L.A., Alexander J., Roberts C.W. (2006). Ocular toxoplasmosis: In the storm of the eye. Parasite Immunol..

[18] Rudzinski M., Argüelles C., Couto C., Oubiña J.R., Reina S. (2019). Immune Mediators against Toxoplasma Gondii during Reactivation of Toxoplasmic Retinochoroiditis. Ocul. Immunol. Inflamm..

[19] Sette A., Crotty S. (2021). Adaptive immunity to SARS-CoV-2 and COVID-19. Cell.

[20] Gajurel K., Dhakal R., Montoya J.G. (2015). Toxoplasma prophylaxis in haematopoietic cell transplant recipients: A review of the literature and recommendations. Curr. Opin. Infect. Dis..

[21] Pradhan E., Bhandari S., Gilbert R.E., Stanford M. (2016). Antibiotics versus no treatment for toxoplasma retinochoroiditis. Cochrane Database Syst Rev..

[22] Kim S.J., Scott I.U., Brown G.C., Brown M.M., Ho A.C., Ip M.S., Recchia F.M. (2013). Interventions for toxoplasma retinochoroiditis: A report by the American Academy of Ophthalmology. Ophthalmology.

